# Patient equity and respiratory syncytial virus Immunoprophylaxis

**DOI:** 10.1186/s13584-019-0288-6

**Published:** 2019-01-28

**Authors:** H. Cody Meissner

**Affiliations:** Tufts University School of Medicine, Boston, MA 02111 USA

**Keywords:** Respiratory syncytial virus, Palivizumab, Immunoprophylaxis, Bronchiolitis

## Abstract

An analysis of benefit and cost is critical for independent advisory groups that provide evidence-based recommendations. In many countries, the role of RSV immunoprophylaxis for infants at increased risk of hospitalization is controversial because of limited benefit and high cost. The report by Ginsberg and co-workers provides evidence, that in Israel, despite the potential benefit of palivizumab prophylaxis in reducing a small number of RSV hospitalizations but no evidence of long-term benefit, the cost is difficult to justify. Ideally, a safe and effective RSV vaccine or more effective and less expensive monoclonal antibody soon will become available.

Limited financial resources dictate that access to unrestricted health care without consideration of cost is no longer possible. The pressing issue is how to address financial stewardship in a fair and equitable manner and how to determine who will and who will not receive a specific intervention. Clinical decisions should not be made on cost alone, but resources used for one patient will not be available for other interventions for other patients who may derive greater benefit for less cost. The question of which interventions should be provided and which interventions should be restricted will become increasingly controversial.

The timely study by Ginsberg and coworkers published in this issue of the *Journal* offers a fresh perspective regarding the issue of benefit and cost from respiratory syncytial virus (RSV) immunoprophylaxis for Israeli infants at increased risk of RSV hospitalization [1]. The authors provide a cost-utility analysis regarding monthly RSV palivizumab prophylaxis to reduce the risk of hospitalization. Epidemiologic, demographic, health service utilization and economic data from the Ministry of Health’s National Hospitalization Database as well as published data are used to determine the net cost per averted disability adjusted life year. Their model includes the costs, resultant treatment savings and improvements in quality of life over a 100-year time period from palivizumab use. Cost effectiveness is defined as less than three times the per capita gross domestic product. The authors conclude, “For all the groups, RSV immunoprophylaxis is clearly not cost effective.” This conclusion is consistent with results from studies from other countries, using different approaches for the economic analyses [2–7].

Some economic analyses of palivizumab cost and benefit have reached a different conclusion, finding prophylaxis to be cost effective or even cost saving. Different conclusions result mainly from different base case assumptions, such as baseline hospitalization rates among children in different risk groups, reduction in hospitalization rates among prophylaxis recipients, the cost of hospitalization, the number of monthly doses administered, the weight of the infant who receives prophylaxis and the acquisition cost and administration fee of palivizumab. Almost all publications supporting the use of immunoprophylaxis come from company employees or consultants or recipients of research funding or other compensation from the company [8]. While this by itself does not indicate bias, a Cochrane review of this subject noted that studies “sponsored by the industry support the cost-effectiveness of palivizumab while practically all studies that were not sponsored by the industry suggest that palivizumab was not cost effective [4].”

The burden of viral respiratory tract disease among children in the first years of life in both developed and less developed countries of the world exceeds that of most other pediatric illnesses [9]. In developed countries, seasonal RSV infections may account for more than one half of all hospitalizations in the first 12 months of life [10]. Because of the high cost of immunoprophylaxis, the limited reduction in RSV hospitalization rates among recipients of prophylaxis and no demonstrable downstream benefit from avoidance of RSV hospitalization, the question emerges as to how much should society be willing to pay to avoid one RSV hospitalization [11]?

RSV immunoprophylaxis is available with palivizumab, a humanized mouse monoclonal antibody produced by recombinant DNA technology [12]. This antibody is administered intramuscularly and is distributed throughout the body including the airways of the lung. When RSV enters the lung of an infant receiving immunoprophylaxis, the antibody binds to a protein on the surface of the virus and prevents replication. The United States Food and Drug Administration licensed palivizumab in June 1998, largely based on the results of a single clinical trial conducted during the 1996–1997 RSV season [13]. This well conducted, randomized, double blind, placebo controlled trial enrolled 1502 infants with prematurity with or without chronic lung disease, two factors recognized to increase the risk of RSV hospitalization early in life. The primary endpoint of this trial was hospitalization with a documented RSV infection. Results demonstrated a modest, overall 5.8% reduction in RSV hospitalization rates (10.6% in placebo recipients and 4.8% in placebo recipients, *p* < .001).

The results of a second randomized, placebo controlled trial of 1287 infants with hemodynamically significant congenital heart disease were published 5 years after FDA licensure for preterm infants (palivizumab had not previously been licensed for use in children with congenital heart disease) [14]. Results again demonstrated a modest benefit from immunoprophylaxis with an overall reduction in RSV hospitalization rate of 4.4% (9.7% among placebo recipients and 5.3% among palivizumab recipients, *p* = .003).

Many observers consider a 4 to 6% reduction in RSV hospitalization rates between placebo and prophylaxis groups as desirable but limited. A second consideration regarding the benefit of RSV prophylaxis is a possible long-term effect, such as a reduction in subsequent airway disease or a reduction in RSV mortality rates. The observation is well established that severe RSV disease early in the first year of life is associated with higher rates of wheezing and asthma in the first decade of life than occurs among children who do not experience severe RSV disease [15]. The perplexing and unresolved question is whether this association is causal and attributable to direct damage to the lung caused by the virus [16]. An alternative theory proposes the association of RSV lower respiratory tract infection and subsequent episodes of asthma and wheezing may reflect a common predisposition. That is, the same anatomic or immunologic abnormalities that predispose to asthma also predispose to severe RSV disease. In this latter scenario, even if a severe RSV infection is avoided by use of immunoprophylaxis, the underlying predisposition to asthma still will exist and avoiding RSV infection will not reduce episodes of wheezing.

If immunoprophylaxis eventually is determined to reduce episodes of wheezing and asthma, this will be an important patient outcome and an important consideration in an analysis of cost and benefit. Several studies have attempted to address this critical question of RSV and wheezing but difficulties in trial design render results inconclusive [17]. A controlled, randomized study of term infants using an investigational second-generation monoclonal antibody (motavizumab) involving healthy term Native American infants found no difference in the rates of medically attended events for asthma over 3 years even though prophylaxis with this investigational drug resulted in an 87% relative reduction in RSV hospitalizations [18]. Another blinded clinical trial randomized preterm infants to palivizumab prophylaxis or placebo and found no statistically significant reduction in episodes of asthma at 6 years of age between groups [19]. Thus, reduction in asthma as a consequence of avoidance of RSV infection secondary to prophylaxis should not be considered in a robust cost analysis. In addition, as Ginsberg et al. note, even if a reduction in wheezing is attributed to palivizumab use, the results of the cost-utility analysis still are not favorable.

Favorable cost analyses of a specific therapy may be driven by a reduction in mortality rates because of future productivity gains over the expected lifetime of the patient. However, neither of the two prospective, randomized, controlled trials involving a total of 2788 infants had sufficient power to demonstrate a statistically significant reduction in RSV morality as a benefit of immunoprophylaxis [13, 14]. Therefore, avoidance of RSV related-mortality cannot reliably be attributed to immunoprophylaxis based on available evidence.

Based on current understanding, the direct cost benefit of immunoprophylaxis is driven solely by savings from a reduction in RSV hospitalization. The cost of monthly immunoprophylaxis from a societal perspective includes drug acquisition, administration fee and drug wastage. The indirect cost benefits from avoidance of RSV hospitalization include caregiver absences from work and out of pocket expenses. Reduction in use of outpatient resources among infants who receive immunoprophylaxis is plausible but this benefit was not evaluated in either of the two randomized trials with palivizumab, so the answer remains uncertain. Because placebo controlled trials after licensure of palivizumab are not possible, it has been and will continue to be difficult to evaluate the true benefit of prophylaxis, especially as trends in the management of outpatient RSV disease evolve rapidly [20].

The American Academy of Pediatrics first issued recommendations for use of palivizumab in 1998 [21]. Since the initial guidelines were published, subsequent iterations have become increasingly restrictive. These changes in recommendations for prophylaxis eligibility evolved with greater understanding of RSV seasonality and geographic distribution (based on CDC data reported to the National Respiratory Virus and Enteric Virus Surveillance System) [22]. Additionally, identification of subgroups of infants truly at greater risk of RSV hospitalization (and therefore more likely to benefit from prophylaxis) has become available. Further information regarding the pharmacokinetics of palivizumab became available in 2012 [23]. Together, these observations have enabled greater precision regarding selection of those infants and young children most likely to derive some benefit from immunoprophylaxis.

## Conclusion

As health care workers and parents await the availability of a safe and effective RSV vaccine or a second generation, longer half-life monoclonal antibody that proves to be more protective, more durable and less costly than immunoprophylaxis with palivizumab, palivizumab likely will remain the only licensed intervention. In the meantime, cost analyses conducted by independent investigators likely will continue to show the high cost of palivizumab prophylaxis with only modest benefit. Based on the analysis by Ginsberg and co-workers, policy-makers in Israel would be justified in restricting the use of palivizumab.

## Abbreviations

CDC: Centers for Disease Control and Prevention
FDA: Food and Drug Administration
RSV: respiratory syncytial virus
